# A Novel Cognitive Behavioral Therapy–Based Generative AI Tool (Socrates 2.0) to Facilitate Socratic Dialogue: Protocol for a Mixed Methods Feasibility Study

**DOI:** 10.2196/58195

**Published:** 2024-10-10

**Authors:** Philip Held, Sarah A Pridgen, Yaozhong Chen, Zuhaib Akhtar, Darpan Amin, Sean Pohorence

**Affiliations:** 1 Department of Psychiatry and Behavioral Sciences Rush University Medical Center Chicago, IL United States

**Keywords:** generative artificial intelligence, mental health, feasibility, cognitive restructuring, Socratic dialogue, mobile phone

## Abstract

**Background:**

Digital mental health tools, designed to augment traditional mental health treatments, are becoming increasingly important due to a wide range of barriers to accessing mental health care, including a growing shortage of clinicians. Most existing tools use rule-based algorithms, often leading to interactions that feel unnatural compared with human therapists. Large language models (LLMs) offer a solution for the development of more natural, engaging digital tools. In this paper, we detail the development of Socrates 2.0, which was designed to engage users in Socratic dialogue surrounding unrealistic or unhelpful beliefs, a core technique in cognitive behavioral therapies. The multiagent LLM-based tool features an artificial intelligence (AI) therapist, Socrates, which receives automated feedback from an AI supervisor and an AI rater. The combination of multiple agents appeared to help address common LLM issues such as looping, and it improved the overall dialogue experience. Initial user feedback from individuals with lived experiences of mental health problems as well as cognitive behavioral therapists has been positive. Moreover, tests in approximately 500 scenarios showed that Socrates 2.0 engaged in harmful responses in under 1% of cases, with the AI supervisor promptly correcting the dialogue each time. However, formal feasibility studies with potential end users are needed.

**Objective:**

This mixed methods study examines the feasibility of Socrates 2.0.

**Methods:**

On the basis of the initial data, we devised a formal feasibility study of Socrates 2.0 to gather qualitative and quantitative data about users’ and clinicians’ experience of interacting with the tool. Using a mixed method approach, the goal is to gather feasibility and acceptability data from 100 users and 50 clinicians to inform the eventual implementation of generative AI tools, such as Socrates 2.0, in mental health treatment. We designed this study to better understand how users and clinicians interact with the tool, including the frequency, length, and time of interactions, users’ satisfaction with the tool overall, quality of each dialogue and individual responses, as well as ways in which the tool should be improved before it is used in efficacy trials. Descriptive and inferential analyses will be performed on data from validated usability measures. Thematic analysis will be performed on the qualitative data.

**Results:**

Recruitment will begin in February 2024 and is expected to conclude by February 2025. As of September 25, 2024, overall, 55 participants have been recruited.

**Conclusions:**

The development of Socrates 2.0 and the outlined feasibility study are important first steps in applying generative AI to mental health treatment delivery and lay the foundation for formal feasibility studies.

**International Registered Report Identifier (IRRID):**

DERR1-10.2196/58195

## Introduction

### Background

The use of digital mental health tools to augment traditional psychotherapies is becoming increasingly important due to a growing shortage of clinicians and other barriers to care (eg, cost and accessibility) [1-3]. Researchers have suggested that digital interventions can increase accessibility and reduce barriers to mental health care, offering flexible and scalable solutions [4,5]. To date, most of the existing digital mental health tools have relied on rule-based algorithms to provide mostly prescripted responses, at times resulting in these tools feeling less natural compared with dialogues with human therapists [6]. The ability of large language models (LLMs) to mimic human language provides the opportunity to build digital mental health tools that may feel more natural and engaging [7,8]. Building on recent advances relating to LLMs, our goal was to create and examine the feasibility of a generative artificial intelligence (AI) tool that can complement traditional cognitive behavioral therapies (CBTs) by facilitating a core therapeutic intervention: Socratic dialogue [9,10]. Developing the tool as a complement to traditional CBT allows it to fulfill basic functions, as other important therapeutic factors, such as other interventions or working alliances, can still be received through therapy. Given the current limitations of LLMs [7], our tool, Socrates 2.0, was designed to ultimately be used in conjunction with a licensed clinician to make the out-of-session CBT practice of evaluating one’s thoughts more engaging compared with traditional worksheets. In this paper, we first describe our process of designing and completing the initial testing of Socrates 2.0, a multiagent tool built using LLMs. We then describe the protocol for a feasibility study to gather qualitative and quantitative data about users’ and clinicians’ experience of interacting with the tool.

### Clinical Background

CBTs have been shown to be effective for a multitude of mental health disorders, including posttraumatic stress disorder, depression, and anxiety, among others [11]. A key intervention in CBTs is Socratic dialogue [9,10,12-15]. Although used in a wide variety of contexts beyond psychotherapy, the Socratic dialogue process therapists use is a collaborative approach to identifying and dismantling patients’ unrealistic or unhelpful beliefs that lead to distress and the expression of psychopathology [9,10,12]. For example, in trauma-focused CBTs, such as cognitive processing therapy [12], common beliefs voiced by trauma survivors center on the perceived ability to have changed the outcome via different actions, such as “If I had dressed differently, I would not have been assaulted.” The cognitive behavioral therapist’s role using Socratic dialogue is to help the patient evaluate the specific belief in the context of the situation to which the belief refers, explore the factual support for the specific belief given relevant circumstances, and explore more realistic and helpful alternative beliefs [12].

Out-of-session practice (ie, homework) is common in CBTs and is often facilitated via worksheets that guide patients to identify, explore, and potentially challenge their thoughts using static Socratic questions. Although research demonstrates that learning and practicing skills via repeated practice and homework is important for symptom improvement [16-20], therapists frequently observe low homework completion rates in practice [21,22]. Possible reasons may include the challenging and noninteractive nature of the worksheets, the time it takes to complete them, and avoidance due to symptoms. We viewed the Socratic process as a good specific use case for an LLM-based tool that guides patients through this process. Given the advanced conversational abilities of LLMs [7], we hypothesized that the use of a tool such as Socrates 2.0 to facilitate Socratic dialogue could be feasible.

### Design and Data Safety Considerations

**Figure 1:**
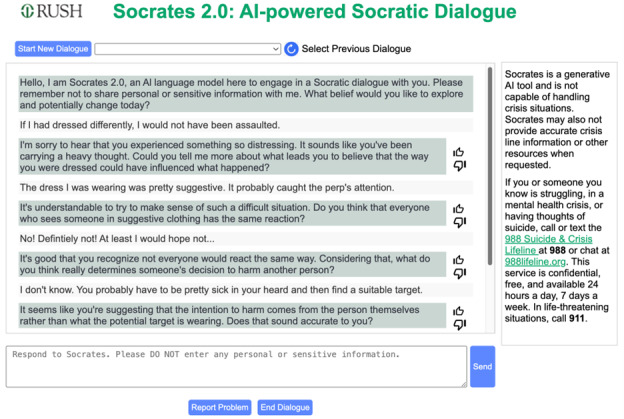
Socrates 2.0 user interface and dialogue example.

To enhance user accessibility, the team prioritized ease of use in the design of Socrates 2.0. The aim was to create an intuitive interface, minimizing the need for therapists to extensively instruct patients on using the tool. After a straightforward log-in process, where users are reminded of the tool’s limitations and prompted for their unique log-in credentials, the interface adopts a familiar chat layout. As shown in Figure 1, the initial interaction features Socrates introducing itself as an AI tool, preemptively clarifying its nonhuman nature. It then invites users to provide a belief they would like to explore or potentially change, a concept readily understandable within the context of CBTs.

Recognizing LLMs’ tendency to generate inaccurate content (hallucinations), information about this limitation and emergency contact details are prominently displayed (Figure 1). This constant visibility of the safety information and AI limitations ensures users are always aware and have necessary resources readily available during their interaction with Socrates. Additional design elements include a reminder in the text entry field against sharing personal information. Socrates 2.0 runs on our own instance of Microsoft Azure (Microsoft Corp) GPT4o (OpenAI) in the Road Home Program’s dedicated resource group and Rush University Medical Center subscription. This is different from a public instance, such as simply connecting to ChatGPT via an application programming interface. The Microsoft Azure (Microsoft Corp) GPT4o (OpenAI) models are stateless, and no data (ie, user prompts or model-generated responses) are stored in the model. Moreover, none of the input or output data of the model or embedding and training data are used by other parties (eg, corporations and researchers). Socrates 2.0 was reviewed by our hospital’s cybersecurity team and is fully compliant with Health Insurance Portability and Accountability Act and our hospital’s data privacy policy. All user data are transferred through secure and encrypted https. All user data are always encrypted and then stored in databases. Users are also encouraged to refrain from providing personally identifiable information or personal health information when they log into Socrates 2.0’s web interface to further decrease any potential risks.

### Socrates 1.0

To create the first version of the tool, Socrates 1.0, the team initially used a zero-shot approach [23], prompting a single-agent model to engage the user in Socratic dialogue. Prompt engineering was facilitated by mental health treatment experts on our team with support from AI engineers. The resulting model was able to facilitate Socratic dialogue, but we noticed several issues, including model responses being overly elaborate or verbose, detracting from the natural flow of a therapeutic conversation. As exchanges became longer, Socrates 1.0 would also forget its role and get off task. We also noticed conversations would become cyclical and the model would get stuck in conversational loops. In addition to challenges with keeping conversations focused, it also became apparent that Socrates 1.0 struggled with determining when a user’s belief had been explored or changed sufficiently to end the dialogue. Although human therapists do this relatively intuitively, monitoring session progress (ie, the extent to which a belief changes throughout the dialogue) and making decisions based on the progress (or lack thereof) are relatively advanced skills.

### Socrates 2.0

**Figure 2:**
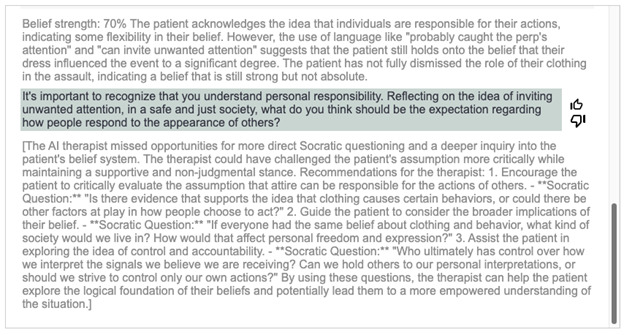
Socrates 2.0 artificial intelligence (AI) external rater and AI supervisor feedback. The first gray statement is from the AI external rater. The second gray statement in brackets is from the AI supervisor. Both responses are hidden from the user; this is a screenshot for illustrative purposes from the developer mode of Socrates 2.0.

**Figure 3:**
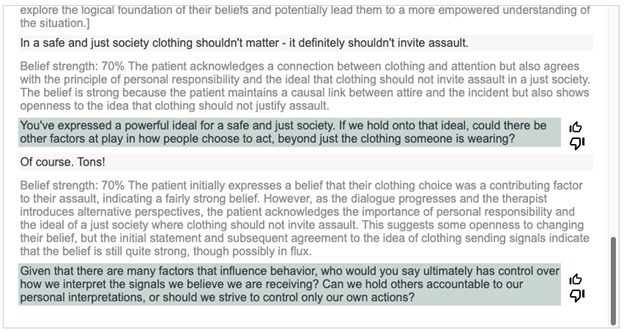
Socrates 2.0 integration of supervisor feedback. The gray statement without brackets is from the artificial intelligence (AI) external rater. AI external rater responses are hidden from the user; this is a screenshot for illustrative purposes from the developer mode of Socrates 2.0.

To mitigate the aforementioned issues, we drew from real-world therapeutic processes and supervision experiences. We created a multiagent tool [24] by adding an AI supervisor and an AI external rater, which were designed to support the AI therapist in facilitating the dialogue without being visible to the user. All AI agents are based on GPT4o available through Microsoft Azure (Microsoft Corp); GPT4o was not fine-tuned for Socrates 2.0 (although GPT4o was used for Socrates 2.0, it is possible that similar performance could have been achieved using other available LLMs). The AI supervisor was designed to monitor the dialogue between the AI therapist and the user and provide real-time feedback to the AI therapist on how to improve the Socratic dialogue and keep the conversation focused. This process is similar to the bug-in-ear supervision method where human supervisors observe therapists’ sessions and provide feedback in real time via a device in the therapists’ ear [25]. Once the AI therapist receives the AI supervisor’s feedback, it will integrate some of this feedback into future responses. Figure 2 shows the AI supervisor feedback in response to a dialogue and is taken from the developer version of Socrates 2.0, which differs from the production version in that it displays the external rater and supervisor feedback. In this example, following the monitoring of the ongoing exchange between the user and the AI therapist, the AI supervisor provides 3 concrete suggestions for improvement. As shown in Figure 3, the AI therapist’s subsequent message incorporates the first of the 3 specific AI supervisor suggestions. By immediately incorporating AI supervisor feedback, the AI therapist can adjust the focus of the dialogue which can help improve the overall Socratic dialogue quality.

The AI external rater, which was designed to assess the dialogue progress by estimating the user’s belief strength, is shown in Figures 2 and 3. When provided to the AI therapist, this information appears to aid in determining when the AI therapist needs to change the types of questions, such as in the case of an original belief not changing in strength or concluding sessions when the user’s beliefs have meaningfully improved. Compared with Socrates 1.0, which did not include the AI external rater, the addition of the AI external rater appeared to drastically reduce the likelihood that the AI therapist would engage in infinite Socratic dialogue by providing belief change rating estimates.

**Figure 4:**
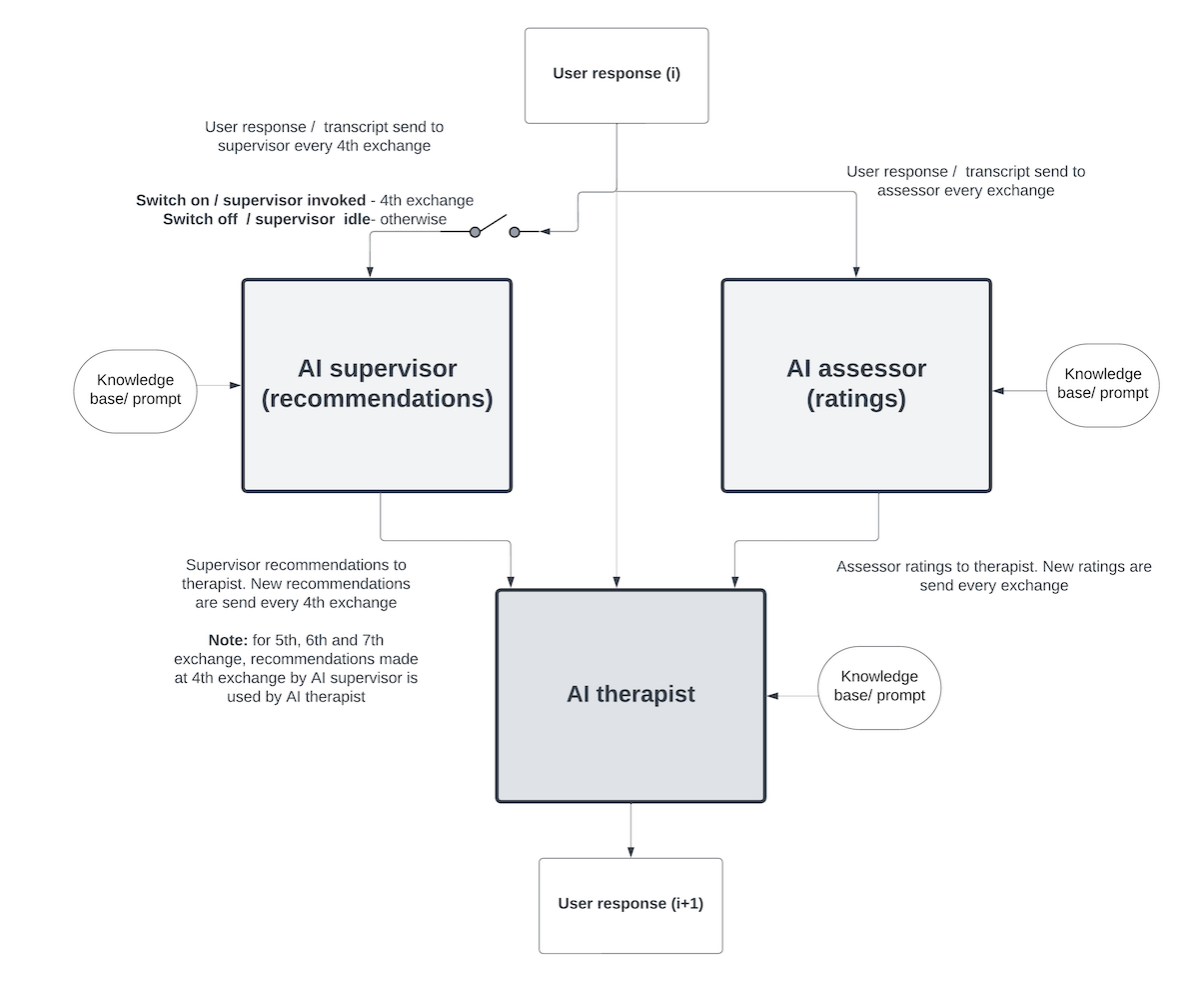
Socrates 2.0 artificial intelligence (AI) entity interactions.

The interaction between the 3 AI agents is detailed in Figure 4. The average response time is approximately 2.11 seconds. To improve the experience and reduce wait times, the user sees the AI therapist respond as soon as it becomes available, even if the AI supervisor response, which takes approximately 7.69 seconds to generate, is still processing. The addition of multiple collaborative AI agents, which sets Socrates 2.0 apart from the original version, appeared to meaningfully impact the tool’s behavior. Iterative prompt engineering was needed and used to improve the individual agent’s behavior and how they worked together. For example, one key and somewhat comical issue the team needed to resolve via prompt engineering was LLM agents conversing with one another (eg, thanking each other for the valuable insights they each provided) in front of the user. Through the inclusion of multiple AI agents [24], CBT experts on our team felt as though Socrates 2.0 produced higher quality Socratic dialogue which seemed to resemble responses from a human therapist more closely.

### Initial Testing

Considering some of the known issues associated with LLMs, a key goal for our team was to ensure that Socrates 2.0 would not only provide users with a solid Socratic dialogue experience but also that it would not provide harmful responses [7]. This latter part was particularly important given its intended use in the context of mental health care. Our team’s internal testing process involved engaging the AI in a diverse range of dialogues to assess the quality and appropriateness of its responses. In the absence of standardized methods to evaluate the response quality of generative AI tools in the context of mental health care, our team tested Socrates 2.0 across >500 conversational scenarios aimed at provoking potentially harmful responses, including the promotion of self-harm and harm to others, sexist and racist remarks, as well as other inappropriate therapeutic behaviors, such as flirting, among many other scenarios. The results were promising, showing that Socrates 2.0 generally avoided such responses. In extremely rare cases (<1%, n=1800 of test cases) where the tool was able to be triggered to provide undesired responses, the AI supervisor quickly (ie, usually within 3-4 exchanges) and effectively redirected conversations away from inappropriate content, further highlighting the benefit of multiagent tools.

### Initial User Feedback

We first gave access to Socrates 2.0 to 6 trained clinicians at our academic medical center who are well-versed in Socratic dialogue. These clinicians had varying degrees of knowledge and comfort with AI tools for mental health. After engaging with Socrates 2.0, members of the study team elicited their feedback on the quality of the Socratic dialogue using a brief semistructured interview which asked about their overall impressions and potential usefulness when combined with clinical care and asked whether they could envision using the tool in their practice. Overall, clinicians were impressed with the quality of the interactions and found them comparable to that of a real-life clinician. Most (5/6, 83%) clinicians stated that they would consider using Socrates 2.0 with their patients, although it is important to recognize that these are clinicians who are used to adopting novel research-based tools and techniques.

We also elicited feedback from a community advisory board (CAB) made up of 6 members with lived experience of mental health disorders and CBTs. After having unlimited access to Socrates 2.0 for 1 week, study team members elicited qualitative feedback during a scheduled CAB meeting, by asking questions, such as “What was your overall impression of using Socrates 2.0?” and “What did you like or not like about engaging with Socrates?” The CAB’s response to Socrates 2.0 was found to be overwhelmingly positive. They saw great value in being able to connect with the AI therapist at any time from anywhere:

I really like how it’s always there- even in the middle of the night.

Some specifically liked that it was not a real therapist, noting the following:

I feel like I can tell [Socrates 2.0] things I wouldn’t say to a human therapist. I like feeling anonymous and it’s non-judgmental.

The CAB found the tool more engaging than common CBT worksheets and suggested they would be more likely to complete out-of-session practice assignments if it involved using Socrates 2.0. User data from that week showed that CAB individuals engaged with Socrates multiple times, with some users using Socrates for up to an hour at a time. The CAB also provided feedback on changes they would like to see with Socrates 2.0, many of which were cosmetic and accessibility-related changes (“I’d like the option to customize my experience, like changing font size, colors, and the background,” “I want to be able to use voice rather than text”), which are currently in development for future versions of the tool.

### Feasibility Study

Following the initial development of Socrates 2.0 and the obtained data from initial users, we devised a feasibility study to formally gather data about users’ experience of interacting with the tool. The goal is to gather feasibility and acceptability data from both patients and clinicians, as information from both groups is critical to inform the eventual implementation of generative AI tools, such as Socrates 2.0, in mental health treatment. We designed this study to better understand how users engage with the tool, including the interaction frequency, length, and time, users’ satisfaction with the tool overall as well as the quality of each dialogue and individual responses. Through the feasibility study, we also plan to obtain information about the ways in which the tool should be improved before it is tested in trials designed to examine the efficacy of Socrates 2.0.

## Methods

### Study Design

The study will use a mixed methods design, combining quantitative and qualitative methods to assess the feasibility of Socrates 2.0 from the perspective of users (ie, potential patients) and clinicians. The decision to obtain feedback from both users and clinicians was made so that our team could better understand how users would perceive the tool and to understand how clinicians could see integrating such a tool into their treatment. The mixed methods approach enables a comprehensive evaluation of the tool from multiple perspectives, providing quantitative feasibility, acceptability, satisfaction, and use of data for statistical analysis, as well as rich contextual data for a qualitative analysis of users’ experiences and perceptions. The combination of quantitative and qualitative data captured throughout this study will be used to make further improvements to Socrates 2.0.

Individuals who consent will be asked to complete brief semistructured interviews and surveys before and after using Socrates 2.0. Users will be granted access to Socrates 2.0 for 4 weeks; clinicians will be granted free unlimited access to Socrates for 2 weeks. During the access period, individuals will be asked to use the tool at their convenience and as often as they wish. To maintain confidentiality, a unique identifier (ie, a combination of random numbers and letters) will be assigned to each participant. During the informed consent process, participants are informed of the potential risks, including loss of confidentiality if their dialogues were to be obtained by someone other than investigators. To help mitigate this risk, in addition to using unique identifiers that only the study team could link to the participant, users are reminded to not give Socrates 2.0 any identifying information about themselves when using the tool to further protect their identity.

### Study Setting

The study will be conducted through the Road Home Program: National Center of Excellence for Veterans and Their Families at Rush University Medical Center in Chicago, Illinois. All qualitative interviews will be conducted via approved and secure video technology (eg, Microsoft Teams; Microsoft Corp), and quantitative feasibility, acceptability, and satisfaction data will be obtained via REDCap (Research Electronic Data Capture; Vanderbilt University) surveys emailed to participants.

### Recruitment

A total of 100 users and 50 clinicians will be recruited to inform the eventual implementation of generative AI tools, such as Socrates 2.0, in mental health treatment. As Socrates is a web-based application and can be accessed via the internet from anywhere, recruitment is open to all individuals, not just patients and providers at Rush University Medical Center. Participants will be recruited from a variety of sources to ensure a diverse sample. Recruitment will occur via The New Normal, Rush University Medical Center’s website, social media, and word of mouth.

### Eligibility

Interested individuals will be screened for general eligibility and will be provided with an electronic version of the consent form. Individuals will be informed that they can contact the study staff before signing the consent form if they have questions. Individuals are eligible to participate in the study if they are aged ≥18 years and have reading and writing proficiency in the English language at least at the sixth-grade level. As Socrates 2.0 is a web-based application, participants must have access to a stable internet connection and a device, such as a computer, tablet, or smartphone, to use the tool and possess the basic technological skills necessary to navigate the tool effectively. In addition, participants must be willing to test and engage in Socratic dialogue with the tool. Aside from not meeting the inclusion criteria detailed earlier, there are no specific exclusion criteria. By having broader inclusion, we hope to be able to speak about the potential of the tool’s feasibility for broader groups and have a more representative community sample. This information would then lend itself to more targeted and highly controlled studies of specific presenting concerns.

### Measures: Users

#### Overview

Following the consent process, participants will be assessed using a combination of surveys and semistructured interviews before and after their 4-week engagement with Socrates 2.0. In addition, objective data based on their interactions with Socrates will be obtained by the research team. The specific data and timepoints at which the data are collected are detailed subsequently. Users and clinicians are asked to complete slightly different assessments; clinician measures are presented subsequently. Table 1 shows the study measures and timepoints for both the users and clinicians.

**Table 1:** Study measure descriptions and timepoints.

Measure			Timepoint		Purpose
**User**					
	Demographics	Baseline		Characterization of sample	
	Patient Health Questionnaire-4	Baseline and 1-month follow-up		Assessment of depression and characterization of sample	
	Interaction Anxiousness Scale-3	Baseline and 1-month follow-up		Assessment of social anxiety and characterization of sample	
	PTSD^a^ Checklist-4	Baseline and 1-month follow-up		Assessment of traumatic stress symptoms and characterization of sample	
	Obsessive-Compulsive Inventory-4	Baseline and 1-month follow-up		Assessment of obsessive-compulsive tendencies and characterization of sample	
	Washington Early Recognition Center Affectivity and Psychosis	Baseline and 1-month follow-up		Assessment of potential mania or psychotic symptoms and characterization of sample	
	System Usability Scale	1-month follow-up		Assessment of usability	
	Acceptability of Intervention Measure	1-month follow-up		Assessment of acceptability	
	Intervention Appropriateness Measure	1-month follow-up		Assessment of tool appropriateness	
	Feasibility of Intervention Measure	1-month follow-up		Assessment of feasibility	
	mHealth^b^ App Usability Questionnaire	1-month follow-up		Assessment of ease of use, satisfaction, and usefulness	
	Working Alliance Inventory-Short Revised	1-month follow-up		Assessment of feelings of rapport and experiences with a therapist	
	Satisfaction	1-month follow-up		Assessment of satisfaction with overall use of Socrates	
	Engagement rates, completion rates, and API^c^ token use	While using Socrates		Time using Socrates, length of dialogue, and number of tokens used during each exchange	
**Clinician**					
	Demographics	Baseline		Characterization of sample	
	System Usability Scale	2-week follow-up		Assessment of usability	
	Acceptability of Intervention Measure	2-week follow-up		Assessment of acceptability	
	Intervention Appropriateness Measure	2-week follow-up		Assessment of tool appropriateness	
	Feasibility of Intervention Measure	2-week follow-up		Assessment of feasibility	
	mHealth App Usability Questionnaire	2-week follow-up		Assessment of ease of use, satisfaction, and usefulness	
	Satisfaction	2-week follow-up		Assessment of satisfaction with overall use of Socrates	
	Engagement rates, completion rates, and API token use	While using Socrates		Time using Socrates, length of dialogue, and number of tokens used during each exchange	

^a^PTSD: posttraumatic stress disorder.

^b^mHealth: mobile health.

^c^API: application programming interface.

#### Demographic Characteristics

Participants will be asked to complete a brief demographic questionnaire at baseline asking them about their age, gender, sexual orientation, ethnicity, race, education, marital status, employment status, household income, and service member or veteran status. The information will be used to characterize the sample and to determine whether any of the participant characteristics are associated with feasibility, acceptability satisfaction, as well as the ways in which participants engage with Socrates 2.0.

#### Pre- and Post-Socrates 2.0 Mental Health Screening Assessments

Participants will be asked to complete brief validated self-report assessments for various mental health areas at baseline and after their month of access to Socrates 2.0. Mental health screening assessments will be used to characterize the sample, understand how mental health symptoms at baseline may impact engagement with the tool, and explore whether potential mental health symptoms change may be statistically associated with the use of Socrates 2.0. Brief screening assessments were chosen to reduce participant burden. To assess depression and generalized anxiety, participants will be asked to complete the Patient Health Questionnaire-4 [26]. The Interaction Anxiousness Scale-3 [27] will be administered to assess social anxiety. Obsessive-compulsive tendencies will be assessed using the Obsessive-Compulsive Inventory-4 [28]. Traumatic stress symptoms and potential manic or psychotic symptoms will be assessed via the Posttraumatic Stress Disorder Checklist-4 [29] and Washington Early Recognition Center Affectivity and Psychosis screen [30], respectively.

#### Before Engaging With Socrates

Participants will be asked to complete a brief semistructured qualitative interview with trained study staff. The interview is intended to better understand participants’ familiarity with AI tools, mental health apps, and prior engagement with mental health services. Participants will be asked semistructured questions. Each semistructured question may be followed up with encouragers to expand on the answers participants provide. The questions are presented in Textbox 1.

**Semistructured interview questions and encouragers.**

- Have you ever used an AI tool before?
  If yes, what kinds? How often? For what purpose?
- Are you currently using, or have you used apps/tools for mental health before (eg, Headspace, Calm, BetterHelp)?
- Have you ever engaged with mental health services?
  If yes, what kinds?
- Have you ever used an AI tool for mental health services?

Following the semistructured interview, the study staff will provide participants with an overview of Socrates 2.0 and walk them through how to use it. The study staff will ensure that participants can log into Socrates 2.0 and answer any questions they may have about using the tool. If requested by the participant, the study staff can be available to answer questions about the tool following the initial overview. The study staff will follow a brief script when introducing the tool, which encourages participants to use Socrates 2.0 as many times as they would like, at any time of the day. Clinicians will be encouraged to engage with Socrates 2.0 as if they were a patient.

#### While Engaging With Socrates 2.0

Participants can provide “thumbs up” or “thumbs down” ratings for responses that Socrates provides. This is optional and intended for participants to indicate particularly helpful or unhelpful responses.

#### After Engaging With Socrates 2.0

Participants will be able to complete a brief Likert-scale survey about their satisfaction with and the perceived helpfulness at the end of each exchange. Participants will also be provided with an option to provide any open-ended feedback they may want to share with the study team about the interaction.

#### One Month After First Engaging With Socrates 2.0

One month after first engaging with Socrates 2.0, participants will be asked to complete a brief survey containing validated measures to assess their experiences and be asked to participate in a semistructured qualitative interview. To assess participants’ subjective assessment of usability, they will be asked to complete the System Usability Scale [31], which is a valid and widely used 10-item measure scored on a 5-point scale (strongly disagree to strongly agree). The Acceptability of Intervention Measure, Intervention Appropriateness Measure, and Feasibility of Intervention Measure [32] is a validated and widely used 12-item measure scored on a 5-point scale (completely disagree to completely agree) that will be administered to determine the perceived acceptability and feasibility of Socrates 2.0. Participants will also be asked to complete the Mobile Health App Usability Questionnaire [33], which is a validated and widely used 18-item measure scored on a 7-point scale (strongly disagree to strongly agree) that assesses ease of use, satisfaction, and usefulness of mobile apps. Finally, the Working Alliance Inventory-Short Revised [34], which is a validated 12-item measure scored on a 5-point scale (seldom to always or always to seldom) will be used to examine participants’ experiences with their AI therapist. Participants will also be asked to complete a semistructured interview surrounding the questions presented in Textbox 2 to better understand their use and impressions of the tool

**Semistructured interview questions.**

- What are your overall impressions of Socrates?
- How often did you use Socrates?
  What prompted you to use Socrates in general and different times you did?
- What would you change about Socrates? Why?
- How would Socrates be helpful to you or other individuals?
  Why might it not be helpful?
  How likely would you be to use it going forward?
  What would make you more likely to use it?
- Who do you believe could benefit most from Socrates?
- How would you present Socrates to others to make them interested and comfortable with checking out the tool?
- How do you think Socrates fits with mental health care?
- Has working with Socrates changed your perception on the utility of AI tools more broadly? (If so, how?)
- Is there anything else you would like to share that I have not asked you about?

#### Objective Use Metrics

Objective metrics, such as the frequency of use, number of exchanges, duration of interactions, and retention rates will be automatically collected via the tool and used to evaluate the feasibility of Socrates 2.0. Specifically, we will examine engagement rates to determine how often and how long users engage with the tool per session and in total over the study period, as well as determine the time since they last used the tool. We will also assess how often users complete the entire interaction or dialogue process with the tool. Finally, we will track LLM token use for each exchange to help with future cost estimations and performance evaluations.

### Measures: Clinicians

As described earlier, both users and clinicians will be asked to participate. Clinicians will complete the same demographics survey, will have access to the same tool, and will be prompted to answer the same questions while using Socrates 2.0. However, unlike the users, clinicians will only be granted 2 weeks of access to Socrates 2.0. Our team determined that this would likely be sufficient time for them to form opinions and evaluate how the tool may fit with their therapeutic work. The team will obtain information about clinician characteristics, such as their experience (eg, number of years in the field), average caseload, populations with whom they work, and whether they are currently providing any interventions that involve cognitive restructuring or Socratic dialogue, among others. In addition, the team will ascertain the clinicians’ familiarity with AI in mental health care. Using semistructured interviews, clinicians will be asked whether they have used AI tools before and whether they have recommended them to patients as well as which types of patients they believe may benefit the most and least from tools such as Socrates 2.0. The team will also ask clinicians about information they believe would be needed for them to feel comfortable recommending AI tools to patients and how AI tools fit with the clinicians’ treatment philosophies.

Following their 2-week access to Socrates 2.0, clinicians will complete similar measures to the other users, including the System Usability Scale [31], Acceptability of Intervention Measure, Intervention Appropriateness Measure, Feasibility of Intervention Measure [32], and Mobile Health App Usability Questionnaire [33]. Clinicians will not be asked about the perceived working alliance with the AI therapist, as they were instructed to interact with the tool differently than actual users, such as by roleplaying some of their own patients to see what answers Socrates 2.0 provides. The semistructured interviews differ from those of the users as they are intended to generate information about clinicians’ perceptions of the tool, aspects they would like to see changed, and how it could fit with their practice. Many of the questions are repeated from the pretool interview, such as questions surrounding the usefulness of AI tools such as Socrates 2.0 as well as which patients may be most and least likely to benefit from such a tool. Repeating interview questions will also enable the study team to determine whether the clinicians’ perceptions and answers change after they have had the opportunity to engage with Socrates 2.0, as it may be more challenging for individuals to imagine what things could look like before directly interacting with the tool.

### Statistical Analyses

#### Quantitative Data Analysis

Quantitative data such as the data from mental health screening assessments and the validated feasibility measures will be analyzed using appropriate descriptive and inferential statistics. Specifically, we will first characterize both the user and clinician samples based on demographic characteristics and the baseline mental health characteristics of the users. Descriptive statistics will also be used to detail the number of completed exchanges. Completeness of the entire interaction will be based on 2 criteria. Either the user clicks the End Dialogue button [rather than closing the window], which finalizes the dialogue, or the user makes statements during the dialogue that signal the end of the conversation. Examples of the latter include statements such as “Thank you. This was really helpful.” After such statements, Socrates 2.0 generally inquires whether there is anything else that it can assist with. If the user ends the dialogue or closes the window at that point or replies suggesting that no further assistance is needed, the dialogue will be considered complete), responses to the assessments administered after using Socrates 2.0, as well as the overall use frequency, duration, and the use of tokens, among others. Any changes over time, such as for mental health symptoms, will be described via commonly-used effect sizes (eg, Cohen *d*), though it is important to note that this is not an effectiveness study and that the uncontrolled design precludes from determining whether any potential changes may be the result of engaging with Socrates 2.0 or external factors. Appropriate inferential statistics, such as multiple linear regression, will be used to determine whether certain demographic or baseline mental health characteristics were associated with the use of Socrates 2.0. We will also evaluate whether the use of Socrates 2.0 influenced assessment ratings, as it is possible that those who used the tool more regularly may have different perceptions of it than infrequent users.

#### Qualitative Data Analysis

Qualitative data will be analyzed using thematic analysis [35], a method for identifying, analyzing, and reporting patterns within data. The thematic analysis will begin with a meticulous process of data familiarization, where researchers immerse themselves in the data to gain a deep understanding of its content. Following this, data will be systematically coded, with codes representing the smallest units of meaning within the data that are relevant to the research questions. These codes will then be collated into potential themes, which are broader patterns of meaning that emerge from the coded data. Each theme will be reviewed and refined to ensure it accurately reflects the coded data and the overall dataset. The final step involves defining and naming themes, providing a detailed analysis of each theme, and the overall story the data tell about the research questions. All quantitative and qualitative analyses will be performed by approved study staff who have the necessary training.

#### Additional Safety Testing

Similar to previous safety testing, we will review interactions for any inappropriate behaviors, including discriminatory statements (racial, sexual, etc), responses that encourage harm of self or others, responses that elicit potentially identifying information from the participant (eg, information protected under Health Insurance Portability and Accountability Act), as well as general obviously inappropriate therapist behaviors, such as flirting with the patient. In addition, transcripts will be reviewed by trained CBT therapists to evaluate the general appropriateness of the responses to determine whether responses were potentially therapeutically inappropriate.

### Ethical Considerations

The study procedures received approval from the Rush University Medical Center institutional review board (23083108). Each prospective participant will receive an electronic copy of the informed consent and have the opportunity to have any study-related inquiries addressed by members of the research team. Participation is contingent upon completion of the electronic consent process. Individuals who do not meet the study criteria or express a need for additional mental health resources will receive information from a member of the research team and be directed to appropriate support services.

Eligible participants will gain access to and receive an introduction to the tool. During both the informed consent process and the introductory session, participants will be informed that the research team does not actively monitor Socrates 2.0, and they should seek appropriate assistance in case of a crisis. Essential information, such as crisis helplines and instructions to dial 911 or visit the nearest emergency room, are prominently and permanently displayed on the main screen upon logging into Socrates 2.0 (Figure 1).

Before the start of the feasibility study, Socrates 2.0 underwent rigorous security testing by an external firm and was cleared for use by Rush University Medical Center’s cybersecurity team. The results of this testing confirmed the security of the web-based platform, encompassing both access to the tool and the storage of the resultant data. These formal procedures aimed to minimize the risk of potential data breaches or losses. Usernames and passwords, generated by the research team using industry-standard protocols, will be linked to eligible participants via deidentified study participant codes. Access to the tool can be promptly revoked by the research team, if necessary. Through these measures, the team has tried to mitigate risks associated with conducting a feasibility study using a web-based platform.

## Results

There are no results to report as of September 25, 2024. Enrollment started in February 2024 and is expected to continue until February 2025. All (ie, positive and negative) findings from this feasibility study will be presented in papers submitted to peer-reviewed scientific publications relevant to Socrates 2.0. Results will also be shared via presentations at scientific conferences as symposia and posters, as well as via Rush University Medical Center’s website and social media. As of September 25, 2024, overall, 55 participants have been recruited.

## Discussion

### Principal Findings

Socrates 2.0 is one of the first functional multiagent generative AI tools intended to complement existing CBTs by engaging users in Socratic dialogue. The feasibility study will contribute to our understanding of whether and how individuals engage with Socrates 2.0 and whether they find their interactions to be beneficial. Importantly, the feasibility study will also provide important additional insights into the safety of the tool, expanding on previous safety testing. Moreover, this study will help us evaluate whether there are certain scenarios in which Socrates 2.0 does not perform as well as anticipated and will enable us to fine-tune future versions of the tool to improve its performance.

By incorporating feedback from clinicians, this study will provide valuable insights into the potential integration of generative AI tools, such as Socrates 2.0, into existing therapeutic practices. Positive findings could pave the way for subsequent studies evaluating the combined use of Socrates 2.0 and CBTs, potentially enhancing engagement compared with conventional worksheets and facilitating skill practice, leading to improved outcomes. Conversely, if the study yields negative results, indicated by low feasibility or satisfaction ratings or significant safety concerns, the feedback will be used to refine the tool further.

### Limitations

The feasibility study has several limitations. First, although Socrates 2.0 is intended for use in mental health contexts, it is open to all users, not just patients with current or former mental health issues. This broad inclusion may impact feasibility, acceptability, and satisfaction ratings, potentially varying between those with and without diagnosed mental health conditions. To address this, we will administer screening assessments to gather mental health information and explore potential associations between symptom severity and feasibility ratings. Second, while Socrates 2.0 is primarily intended to complement CBTs by facilitating cognitive restructuring, this feasibility study initially evaluates it as a stand-alone tool. Using Socrates 2.0 as a complement to CBT enables the tool to fulfill a limited functionality (ie, engaging users in Socratic dialogue), while other core therapeutic elements, including working alliance and other therapeutic techniques, can still be received through the sessions with the human clinician. Thus, the obtained feasibility results from using Socrates 2.0 in isolation may differ compared with those that would be obtained following the integration with psychotherapy. Third, the study’s description explicitly mentions testing a mental health–focused generative AI tool, which might deter users and clinicians less receptive to this novel technology from participating. Consequently, this self-selection bias could skew the findings related to feasibility, acceptability, and satisfaction. Finally, Socrates 2.0 was purposefully designed to facilitate Socratic dialogue. Therefore, the insights gleaned from this study will not be universally applicable to other generative AI–based mental health tools designed for different purposes.

### Conclusions

The development of Socrates 2.0 marks a significant advancement in the use of generative AI in the context of CBT and mental health care more broadly. By combining multiple collaborative AI agents, Socrates 2.0 facilitates Socratic dialogue on a range of beliefs while minimizing common LLM issues such as looping. Initial positive user and clinician feedback suggests that generative AI tools, such as Socrates 2.0, could be feasible. However, formal studies, such as the feasibility study described in this paper, and tests of tools, such as Socrates 2.0 in conjunction with CBT are needed to better understand the true feasibility, as well as the potential risks and problems of generative AI tools as complements to CBT. Future studies should examine whether using LLMs would make out-of-session practice, such as examining one’s thoughts, more engaging for patients than worksheets [21]. It should also be formally tested whether a generative AI tool would be able to engage patients in Socratic dialogue at least as well as a newly trained therapist, such as by providing therapists with transcripts and comparing the quality of subsequent human responses to those provided by AI. Finally, future studies should formally assess digital literacy, which could be an important factor that explains engagement with tools, such as Socrates 2.0. The outlined feasibility study will provide user and clinician feedback that can be used to evaluate the tool and further refine it for broader application and testing its efficacy with psychotherapy. We hope that the description of Socrates 2.0 and the associated feasibility trial will serve as a valuable model for other researchers and developers aiming to harness the power of AI in mental health [7].

## Abbreviations

AI: artificial intelligence
CAB: community advisory board
CBT: cognitive behavioral therapy
LLM: large language model
REDCap: Research Electronic Data Capture

## Data Availability

Data sharing is not applicable to this paper as no datasets were generated or analyzed.
